# Improving Appropriate Use of Antifungal Medications: The Role of an Over-the-Counter Vaginal pH Self-Test Device

**DOI:** 10.1080/10647440300025523

**Published:** 2003

**Authors:** Subir Roy, James C. Caillouette, Joel S. Faden, Tapon Roy, Diana E. Ramos

**Affiliations:** 1 Professor Obstetrics and Gynecology Keck School of Medicine of the University of Southern California Women’s and Children’s Hospital 1240 N. Mission Road, Room L1022 Los Angeles CA 90033 USA; 2 Private Practice Obstetrics and Gynecology (Retired) Pasadena CA USA; 3 FDA Regulatory Consultant Washington, DC USA; 4 Statistical Consultant Bethel CT USA; 5 Private Practice Obstetrics and Gynecology Monterey Park CA USA

## Abstract

*Objectives:* To determine whether patients can understand and use the vaginal pH device in the diagnosis of vaginitis. To compare whether vaginal pH readings determined by patients and healthcare providers are similar. To determine whether vaginalpHcan reduce inappropriate over-the-counter (OTC) antifungal medication use and improve the correct diagnosis of vaginitis.

*Methods:* One hundred and fifty-one women indicated their belief about the cause of their vaginal infection, read the instructions of the vaginal pH device package insert, used the device and interpreted the findings. The patient interpretations were compared with results obtained by healthcare providers, blinded to patient findings.

*Results:* Over 96% of patients stated that they could easily read the instructions, use the vaginal pH device and interpret the readings. They obtained the same readings as healthcare professionals (Kappa = 0.9). Restricting the use of OTC antifungal medications to those individuals with vaginitis symptoms and vaginal pH ≤ 4.5 significantly reduced inappropriate use by approximately 50%, Fisher's exact test,*p*-value = 0.018. Conversely, seeking healthcare provider assessment with vaginal pH > 4.5, leads to correct diagnosis of vaginitis.

*Conclusions:*  The vaginal pH device can be used as an OTC diagnostic tool by consumers when a vaginal infection is suspected. Vaginal pH readings would direct patients whether to purchase an antifungal medication or seek professional diagnosis from a healthcare provider. Understanding and use of this vaginal pH device could reduce inappropriate use of OTC antifungal medications by approximately 50% and improve the correct diagnosis of vaginitis.


*Infect Dis Obstet Gynecol 2003;11:209–216*

### Improving appropriate use of antifungal medications: the role of an over-the-counter vaginal pH self-test device

**Subir Roy[1], James C. Caillouette[2], Joel S. Faden[3], Tapon Roy[4] and Diana E. Ramos[5]**

[1]*Professor Obstetrics and Gynecology, Keck School of Medicine of the University of Southern California, Women's and Children's Hospital, Los Angeles, CA*
[2]*Private Practice Obstetrics and Gynecology (Retired), Pasadena, CA*
[3]*FDA Regulatory Consultant, Washington, DC*
[4]*Statistical Consultant, Bethel, CT*
[5]*Private Practice Obstetrics and Gynecology, Monterey Park, CA*

***Objectives:*** To determine whether patients can understand and use the vaginal pH device in the diagnosis of vaginitis. To compare whether vaginal pH readings determined by patients and healthcare providers are similar. To determine whether vaginal pH can reduce inappropriate over-the-counter (OTC) antifungal medication use and improve the correct diagnosis of vaginitis.
***Methods:*** One hundred and fifty-one women indicated their belief about the cause of their vaginal infection, read the instructions of the vaginal pH device package insert, used the device and interpreted the findings. The patient interpretations were compared with results obtained by healthcare providers, blinded to patient findings.
***Results:*** Over 96% of patients stated that they could easily read the instructions, use the vaginal pH device and interpret the readings. They obtained the same readings as healthcare professionals (Kappa = 0.9). Restricting the use of OTC antifungal medications to those individuals with vaginitis symptoms and vaginal pH $\leq 4.5$ significantly reduced inappropriate use by approximately 50%, Fisher's exact test, $p$-value = 0.018. Conversely, seeking healthcare provider assessment with vaginal pH > 4.5, leads to correct diagnosis of vaginitis.
***Conclusions:*** The vaginal pH device can be used as an OTC diagnostic tool by consumers when a vaginal infection is suspected. Vaginal pH readings would direct patients whether to purchase an antifungal medication or seek professional diagnosis from a healthcare provider. Understanding and use of this vaginal pH device could reduce inappropriate use of OTC antifungal medications by approximately 50% and improve the correct diagnosis of vaginitis.

Key words: AID; SELF-DIAGNOSIS; VAGINITIS

Dr. Caillouette is founder and principal owner of FemTek LLC and Dr. Faden has a financial interest in FemTek LLC, which holds the intellectual property rights to the vaginal pH device used in this study. This study was funded by FemTek LLC.

Correspondence to: Subir Roy MD, Department of Obstetrics and Gynecology, Keck School of Medicine of the University of Southern California, Women's and Children's Hospital, 1240 N. Mission Road, Room L1022, Los Angeles, CA 90033, USA. Email: subirro@usc.edu

© 2003 The Parthenon Publishing Group 

Since the United States Food and Drug Administration (FDA) allowance of over-the-counter (OTC) vaginal antifungal medication in 1990[1], there have been numerous reports of inappropriate use of such therapy[2–4]. These observations are not surprising because of the high likelihood of bacterial infections responsible for vaginitis. Bacterial infections account for 11–64% of vaginal infections in symptomatic non-pregnant patients[5] while trichomonal infections account for 5–25% of family planning or gynecology clinic visits and up to 32% in STD clinics[6]. Physician or healthcare provider use of vaginal pH testing has been shown to be of utility in diagnosing the causes of vaginitis[7–9]. We have shown that subjects can read the package insert and understand the role of a vaginal pH device[10]. It is our suggestion that patients can use the vaginal pH self-test device (Figure 1) as a screening tool, leading to less inappropriate use of antifungal medications and more appropriate use of healthcare provider examinations.

In order to understand better the place of vaginal pH testing some historical facts are reviewed. pH paper has been described to test vaginal pH in an American medical textbook published as early as 1950[11]. The early textbook specifically recommended Nitrazine® (phenaphthazine) pH paper for vaginal pH testing. The same Nitrazine pH paper is used for the vaginal pH self-test device.

Prior to the availability of the vaginal pH device[12], it was the practice of physicians to press a strip of pH paper against the vaginal wall while holding it with either the fingers or a hemostat or to place it in the secretions from the vaginal pool collected in the posterior fornix[6]. The latter practice is not recommended because of contamination with cervical mucus, blood or semen which may result in an incorrect vaginal pH reading[6] (generally greater than 6.0). The vaginal pH device makes it convenient for the physician or patient to obtain a vaginal pH reading from the lateral outer third of the vagina by mounting the paper on the end of a probe, allowing for a less cumbersome procedure and a correct reading.

Secretions from the normal vaginal wall (outer third) of a reproductive-age female are acidic, in the pH range of 3.8–4.5[13]. Vaginal homeostasis is maintained by interrelationships among the endogenous microflora, metabolic products of the microflora and estrogen levels[13,14]. Studies clearly indicate that estrogen is responsible for proliferating surface vaginal epithelium[15,16]. In response to estrogen, the glycogen content within the vaginal cells is increased and released into the vaginal lumen[17], supporting the growth of various strains of $H_2O_2$- and lactic acid-producing lactobacillae, that are critical for producing an acidic pH and maintaining vaginal health[14]. The resulting healthy vaginal microflora is made up of numerous microorganisms including gram-positive and gram-negative aerobic, facultative and obligate anaerobic bacteria[14,18].

An elevated vaginal pH level (greater than 4.5, considered a positive finding) may indicate various

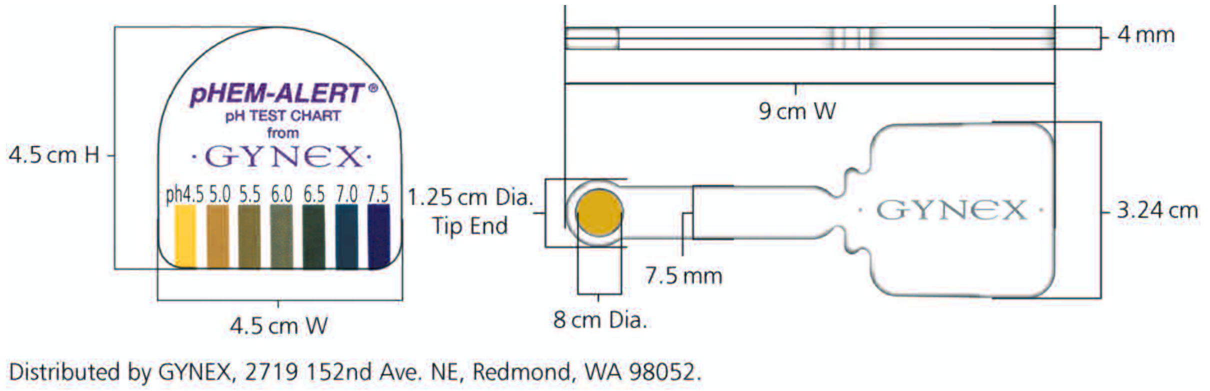

**Figure 1** The OTC version of the pH self-test device is identical to the professional/prescription version, except for the package insert, which has been modified specifically to address the needs of the lay user. Both versions are comprised of a plastic probe with pH paper on one end, a color chart and a package insert. The probe and color chart are illustrated above conditions, including bacterial infections such as bacterial vaginosis (BV), *Trichomonas vaginalis* infections, group B streptocococcus (GBS) or other pathogenic organisms[9]. However, like an elevated temperature, an elevated pH alone is only presumptive and not diagnostic. A diagnosis of the underlying condition or cause of the elevated pH should be established by obtaining additional history, performing tests and observing the woman's signs and symptoms.

Although prescribed for vaginal testing decades before, the seminal work establishing the importance of vaginal pH in the diagnosis of vaginal infections was confirmed by Amsel and co-investigators in the early 1980s. The Amsel criteria remain the cornerstone for differential diagnosis of BV today[7], with vaginal pH alone providing the greatest sensitivity of the four clinical signs, but the lowest specificity[19]. Many clinicians today determine vaginal pH routinely as part of well-woman examinations and as part of the diagnostic process for patients presenting with symptoms of vaginitis[20,21].

This study was conducted to determine whether patients could understand and use a vaginal pH device and interpret test results compared with those made by a healthcare provider, as an aid in the diagnosis of vaginitis. Additionally, could appropriate use and interpretation of a vaginal pH device reduce the numbers of individuals inappropriately using OTC antifungal medication and lead to correct diagnoses of vaginitis?

### SUBJECTS AND METHODS

The study was conducted under the oversight of the IRB Company, Camino de los Mares, CA. Three investigational sites participated in the study: one private practice in North Dakota, one private practice in California and one health clinic in California. These individuals are listed in the acknowledgement section. The patients selected for study were of varied background, educational levels and ages, who were target users (symptomatic patients) and normal controls (asymptomatic patients). As women presented at the participating centers, they were screened to see if they met the inclusion or exclusion criteria. Women who qualified for the study were asked to participate and those who agreed signed informed consents.

The inclusion criteria, for symptomatic women, included vaginal signs or symptoms such as itching, burning, unpleasant odor, unusual discharge and that they have regular menstrual periods. The inclusion criteria for women without symptoms, stipulated that they have regular menstrual periods and be visiting the clinic for routine examination for a non-vaginally related matter. Patients were excluded if they were not mentally or physically capable of reading the instructions and/or performing the test. They were also excluded if they had douched or used contraceptive creams or gels within the last 24 hours; had unprotected sexual intercourse within the last 24 hours; were currently menstruating or the last menstrual period had not been over for 5 days; or were currently pregnant.

Each participant was taken to an examination room and provided a vaginal pH self-test device, the package insert and a patient questionnaire. The patient was then asked to read the package insert, perform the test and complete the questionnaire after performing the test in privacy. Completed questionnaires were immediately given to the study coordinator who kept the results blinded from the patient's healthcare provider (physician or nurse practitioner).

After the patient had finished, the healthcare provider performed a vaginal pH test using the vaginal pH device. A medical examination appropriate to the nature of the patient visit, including microscopy or culture when appropriate, was then carried out. The healthcare provider recorded the test result and completed the study questionnaire without asking the patient any questions regarding her test, its result or her answers to the

**Table 1** Distribution of patients by investigational site and presenting condition

| *Investigational site* | *Condition* | | |
|---|---|---|---|
| | *Normal* | *Symptomatic* | *Total* |
| 1 | 11 | 38 | 49 |
| 2 | 10 | 40 | 50 |
| 3 | 12 | 40 | 52 |
| Total | 33 | 118 | 151 |

questions. The healthcare provider was asked to distinguish between yeast, bacteria or other, in the same manner as we had asked the patients who were not expected to know or diagnose bacterial vaginosis (BV). For practical purposes a diagnosis of 'bacteria' is consistent with BV.

The test results were analyzed using standard statistical methods, including the Fisher's exact test (FET), while the exact permutational version of the Kappa statistic was performed for the extent of agreement between the results of the test when used by patients and healthcare providers using SAS Version 8.01 PROC FREQ[22] and Proc-StatXact 4[23] for SAS.

**Table 2** Demographic characteristics of patients

| *Demographic characteristics* | *Value/number of patients* | *Percentage of patients* |
|---|---|---|
| Age, years | | |
| Average ($n$ = 149) | 34.1 | |
| Minimum | 17 | |
| Maximum | 73 | |
| No response given | 2 | |
| Ethnicity | | |
| Caucasian | 59 | 39 |
| African American | 5 | 3 |
| Hispanic | 80 | 53 |
| Asian American | 2 | 1 |
| Other | 1 | 1 |
| No response given | 4 | 3 |
| Years of education | | |
| 0–12 (actual range) | 87 | 58 |
| 13–16 | 44 | 29 |
| > 16 (graduate) | 3 | 2 |
| No response given | 17 | 11 |

### RESULTS

The distribution of patients by site and presenting condition is provided in Table 1. One hundred and fifty-one women were enrolled in this study. Thirty-three of the 151 women (22%) were normal (asymptomatic) and 118 (78%) were symptomatic. Of the 118 symptomatic patients, 96 were premenopausal, not pregnant, and with a uterus *in situ*. The healthcare provider did not provide a final diagnosis in eight, leaving 88 symptomatic premenopausal patients for whom patient and healthcare diagnoses were available for analysis. Because the assessment of the product's ease of use or the readability of the instructions for use are not influenced by exclusion criteria, the entire population of enrolled patients was used in selected analyses.

The demographics of the enrolled patients are provided in Table 2. The women ranged in age from 17 to 73 years old (average 34.1). The study included women with a diversity of ethnic backgrounds. The educational backgrounds of the women were also diverse ranging from 0 years of formal education to graduate school.

The concordance (represented by a Kappa statistic) between the vaginal pH recorded by the patient and that recorded by the healthcare provider were evaluated separately for all patients and premenopausal patients. The findings were examined considering both the pH readings as discrete values (4.5, 5.0, 5.5, 6.0, 6.5, 7.0, 7.5):

**Table 3** Initial patients' self-diagnoses versus healthcare providers' diagnoses

| | *Healthcare providers' diagnoses* | | | | | |
|---|---|---|---|---|---|---|
| *Patients' initial self-diagnoses* | *Yeast* | *Bacteria/ trichomonas* | *Normal* | *No conclusion or no response* | *Total* | *Subtotal** |
| Yeast | 22 | 13 | 16 | 3 | 54 | 51 |
| Bacteria | 2 | 3 | 1 | 0 | 6 | 6 |
| Yeast and bacteria | 1 | 1 | 0 | 0 | 2 | 2 |
| Other | 0 | 1 | 10 | 3 | 14 | 11 |
| No response given | 3 | 3 | 12 | 2 | 20 | 18 |
| Total | 28 | 21 | 39 | 8 | 96 | 88 |

*The patients for whom the healthcare provider rendered a diagnosis of vaginitis

Kappa for all, 0.891 and for premenopausal, 0.898 and as binary values based around the cut-off of 4.5 (dichomotous: ≤ 4.5 and > 4.5): Kappa for all, 0.870 and for premenopausal, 0.880. The findings demonstrate a high degree of concordance (generally considered 0.8 to 1.0) for all four analyses.

Of the 88 symptomatic premenopausal women who had a diagnosis documented by the healthcare provider, 32% (28/88) were diagnosed with yeast, 23% (21/88) with bacteria or trichomonas and 45% (39/88) were found to be normal (Table 3).

At the time of their enrollment in the study, 51 of the 88 symptomatic premenopausal women believed they had a yeast infection at entry into the study before using the vaginal pH device (58%). Of these 51 women, 43% (22/51) were diagnosed as having a yeast infection, 26% (13/51) as having a bacterial or trichomonas infection and 31% (16/51) were diagnosed as normal by the healthcare provider examination. Therefore, if all 51 women had self-medicated themselves with OTC antifungal medications, 57% (29/51) would have been wrong. Of the 29 who would have misused antifungal medication, 45% (13/29) would have continued to suffer an opportunistic untreated bacterial infection during their course of self-medication.

After performing the vaginal pH test (Table 4), when the test was less than or equal to 4.5, 42% (21/50) thought they had a yeast infection while 76% (16/21) actually had a yeast infection. This represents a significant reduction in misuse of antifungal medications of greater than 50%, from 57% (29/51) to 24% (5/21), FET = 0.018. Of the five patients without a yeast infection, one (20%) would have continued to suffer from an opportunistic bacterial infection. Thus, inappropriate therapy for opportunistic infections would have been reduced from 45% (13/29) to 20% (1/5), FET = 0.38, a non-significant reduction because of small numbers. When the test was greater than 4.5, 13% (5/38) had a yeast infection, as determined by the healthcare provider examination.

In order to determine vaginal pH test performance, standard definitions were used (Table 4)[24]. The information contained in Table 5 was re-formulated into a dichotomous form (Table 6), which permitted the calculation of test

**Table 4** Test performance definitions[24]

| | *Disease positive (+)* | *Disease negative (–)* | *Total* |
|---|---|---|---|
| Test result positive (+) | TP | FP | TP+FP |
| Test result negative (–) | FN | TN | FN+TN |
| Total | TP+FN | FP+TN | All |
| | Sensitivity = TP/(TP+FN) | Specificity = TN/(FP+TN) | |

TP, true positive; FP, false positive; FN, false negative; TN, true negative; positive predictive value (PPV) = TP/(TP+FP); false-positive rate = (100% – PPV); negative predictive value (NPV) = TN/FN+TN); false-negative rate = (100% – NPV)

**Table 5** Patient's response after performing vaginal pH test and healthcare provider's (HCP) diagnosis

| | *HCP: pH > 4.5* | | | | *HCP: pH ≤ 4.5* | | | | |
|---|---|---|---|---|---|---|---|---|---|
| | *Yeast* | *B/T* | *Normal* | *Total* | *Yeast* | *B/T* | *Normal* | *Total* | *Grand Total* |
| Patient's decision after self-test of vaginal pH device: | | | | | | | | | |
| Rx with OTC anti-fungal medications* | 3 | 2 | 4 | 9 | 16 | 1 | 4 | 21 | 30 |
| Contact healthcare provider (HCP)** | 1 | 16 | 6 | 23 | 5 | 0 | 16 | 21 | 44 |
| Do nothing*** | 1 | 2 | 3 | 6 | 2 | 0 | 6 | 8 | 14 |
| Total | 5 | 20 | 13 | 38 | 23 | 1 | 26 | 50 | 88 |

B = bacterial, T = trichomonas; FET = Fisher's exact test (*p*-value): *3/9 versus 16/21 = 0.04, **17/23 versus 5/21 = 0.002, ***3/6 versus 2/8 = 0.58

**Table 6** Patient pH readings versus healthcare providers' diagnoses (dichotomous arrangement)

| | *Healthcare providers' diagnoses* | | |
|---|---|---|---|
| | *Bacteria (B)/trichomonas (T)* | *Yeast or normal* | *Grand total* |
| Patients' pH readings | 20 | 18 | 38 |
| pH > 4.5 | 1 | 49 | 50 |
| pH < 4.5 | 21 | 67 | 88 |
| Total | | | |

**Table 7** Study test performance as a function of various prevalence assumptions and compared with literature estimates

| | *Observed results from this study (Table 6)* | *Assuming a true prevalence of the following* | | | *Composit values from references 7–9* |
|---|---|---|---|---|---|
| Sensitivity | 0.95 | | | | 0.95 |
| Specificity | 0.73 | | | | 0.61 |
| Prevalence | 0.24 | 0.2 | 0.3 | 0.5 | 0.3 |
| Positive predictive value | 0.53 | 0.47 | 0.6 | 0.78 | 0.51 |
| Negative predictive value | 0.98 | 0.98 | 0.97 | 0.94 | 0.97 |

performance for vaginal pH in this study (Table 7). Since the true prevalence of BV in any symptomatic, non-pregnant population is not known (ranging somewhere between 11 and 64%)[5], corresponding to a vaginal pH > 4.5, the positive predictive value (PPV) and negative predictive value (NPV) were also calculated for three additional assumptions for prevalence (0.20, 0.30 and 0.50). These assumptions lie within the range of the reported medical literature[5]. The last column of Table 7 lists the test characteristics of the composite of three studies taken from the literature where healthcare providers obtained vaginal pH and diagnosed the cause of the vaginitis[7–9].

The patient was given a questionnaire to complete after reading the package insert and performing the test. To the question, 'Was the test easy to use?' 146 patients said 'yes', two said 'no' and three did not respond. Assuming a worst-case approach in which the 'absence of a response' is considered a 'no', 97% (146/151) of the women found the test easy to use. To the question, 'Were the instructions easy to follow?' 145 patients said 'yes', three said 'no' and three did not respond. Again assuming a worst-case approach, 96% (146/151) of the women found the instructions easy to follow.

### DISCUSSION

This study demonstrates that the device and labeling are well designed and meet the needs for home testing. Nearly all of the patients found the test easy to use and the instructions easy to follow. A parallel study demonstrated that age, years of education, ethnicity and clinic location did not alter the ability of subjects to read and understand the package insert of the vaginal pH device[10]. Furthermore, the high degree of concordance (0.870 to 0.891) of patients' vaginal pH readings with those obtained by the healthcare provider suggests that the vaginal pH device is designed and labeled appropriately.

This study suggests that by restricting antifungal use to symptomatic patients having vaginal pH ≤ 4.5, would reduce misuse of antifungals by about 50%. Such misuse and overuse of incorrect treatment by symptomatic women is extensively reported in the medical literature[2–4]. According to a survey of 390 gynecologists conducted by the Institute of Epidemiological Research, an estimated 44% of patients diagnosed with bacterial vaginosis (BV) had initially treated themselves with OTC antifungal medications for what they had improperly assumed was a yeast infection[20]. This observation is amplified by a study of patient self-diagnosis in 111 women across the US, in which 67% of women who believed they had a yeast infection were found to be incorrect[25]. Because of the improper use of OTC antifungal medications, the vaginal microflora may be altered[26] and opportunistic infections such as BV may go unchecked and untreated. Vaginitis, in particular BV, has been associated with increased risk of serious obstetric and gynecologic complications and disorders[27–31].

Since the patient is advised to see her doctor/ healthcare provider when the test is positive (pH > 4.5), a false-positive test would only cause the symptomatic patient to visit her healthcare provider for proper diagnosis of an abnormal condition. The probability of a false-negative vaginal pH in a symptomatic patient is relatively low given the specificities reported in the medical literature (and summarized in this document) and supported by the clinical findings reported above. Further, the symptomatic patient who has a negative (pH ≤ 4.5) finding would be in the same position she is today (i.e. she could choose to initiate treatment with an OTC antifungal medication or see her doctor). Since there is no OTC treatment currently available for bacterial and trichomonas infection, the potential gain of reducing the misuse of antifungal medications in the larger number of true-positive and true-negative cases clearly outweighs the risk of a false-negative finding.

It is noteworthy that the test characteristics of the patient self-test of the vaginal pH device were almost exactly the same as those of the composite of three studies taken from the literature[7–9] where healthcare providers obtained vaginal pH and diagnosed the cause of the vaginitis (Table 7). Thus, the test performance characteristics of the vaginal pH self-test device are reasonable and acceptable as an aid in the diagnosis of vaginitis. The correct use of such a device could lead to a more appropriate use of OTC anti-fungal medications and will encourage a more appropriate utilization of healthcare providers. This leads to a proper diagnoses of vaginitis and improved health for women*.

### ACKNOWLEDGEMENTS

We acknowledge the contributions and support of patients recruited from the following sites by the respective individuals and their staff: (1) Private practice: Wendall.Wall, Jr. MD, 275 South 11th Street, Wahpeton, ND 58075; (2) Peter Benson, MD, 8902 Woodman Avenue, Arleta, CA 91331; (3) Private practice: Diana E. Ramos, MD, 880 S. Atlantic Blvd., Suite 302, Monterey Park, CA 91754.

### REFERENCES

1. Food and Drug Administration (FDA) approval of clotrimazole 1% cream and 100 mg inserts on November 30, 1990, Ingredients and Dosages Transferred from Rx-to-OTC Status, FDA's Orange Book: Approved Drug Products with Therapeutic Equivalence Evaluations, October 2002
2. Weisberg M, Summers P. Patient self-diagnosis of vulvovaginal candidiasis. *Female Patient* 1996; 21:60–4
3. Sihvo S, Ahonen R, Mikander H, Hemminki E. Self-medication with vaginal antifungal drugs: physician's experiences and women's utilization patterns. *Fam Pract* 2000;17:145–9
4. Ferris DG, Nyirjesy P, Sobel JD, Soper D, Pavletic A, Litaker MS. Over-the-counter antifungal drug misuse associated with patient-diagnosed vulvovaginal candidiasis. *Obstet Gynecol* 2002;99:419–25
5. French JL, McGregor JA. Bacterial vaginosis: history, epidemiology, microbiology, sequelae, diagnosis, and treatment. In: Borchardt KA, Noble MA, eds. *Sexually Transmitted Diseases:*

*The FDA permitted OTC use of the vaginal pH self-test device in October, 2001 because of the following factors: first, this clinical study demonstrated the validity of the vaginal pH self-test device. Second, the questions that the FDA raised for an OTC device were answered. Third, the use of the vaginal pH self-test device is substantially equivalent to the Professional Version already approved for use.

*Epidemiology, Pathology, Diagnosis, and Treatment.* Boco Raton: CRC Press, 1997:4–39
6. Rein MF, Muller M. *Trichomonas vaginalis* and trichomoniasis. In: Holmes KK, Mardh P-A, Sparling PF, Wiesner PJ, Cates, Jr. W, Lemon SM, Stamm WE, eds. *Sexually Transmitted Diseases*, Second edition, McGraw–Hill, Inc, 1990:481–92
7. Amsel R, Totten PA, Spiegel CA, Chen KCS, Eschenbach DA, Holmes KK. Nonspecific vaginitis. *Am J Med* 1983;74:14–22
8. Eschenbach DA, Hillier SL, Critchlow C, Stevens C, DeRouen T, Holmes KK. Diagnosis and clinical manifestations of bacterial vaginosis. *Am J Obstet Gynecol* 1988;158:819–28
9. Caillouette JC, Sharp CF, Zimmerman GJ, Roy S. Vaginal pH as a marker for bacterial pathogens and menopausal status. *Am J Obstet Gynecol* 1997; 176:1270–7
10. Roy S, Caillouette JC, Faden J, Roy T. The role of an over-the-counter vaginal pH device package insert: can subjects learn what the device is for and how to use it? (submitted for publication)
11. Eastman NJ, *Williams Obstetrics*, 19th edition, New York, Appleton-Century–Crofts, Inc, 1950: 389–91
12. Food and Drug Administration's Premarket Notification [Section 510(k)] clearance to commercially distribute the Professional vaginal pH device, dated May 13,1996
13. Smith KPB. Estrogens and the urogenital tract. Studies on steroid hormone receptors and a clinical study on a new estradiol releasing vaginal ring. *Acta Obstet Gynecol Scand* 1993;72(Suppl 157):S1–26
14. Boskey ER, Telsch KM, Whaley KJ, Moench TR, Cone RA. Acid production of vaginal flora *in vitro* is consistent with the rate and extent of vaginal acidification. *Infect Immun* 1999;67:5170–5
15. Rakoff AE, Feo LG, Goldstein L. The biological characteristics of the normal vagina. *Am J Obstet Gynecol* 1944;47:467–94
16. Lang W. Vaginal acidity and pH: a review. *Obstet Gynecol Surv* 1955;10:546–60
17. Gregoire AT, Kandil O, Ledger WJ. The glycogen content of human vaginal epithelial tissue. *Fertil Steril* 1971;22:64–8
18. Stahl CE, Hill GB. Microflora of the female genital tract. In: Galask RP, Larsen B, eds. *Infectious Diseases in the Female Patient*, New York, Inc: Springer–Verlag, 1986:16–42
19. Hillier SL, Holmes KK. Bacterial vaginosis. In: Holmes KK, Mardh P-A, Sparling PF, Wiesner PJ, Cates, Jr. W, Lemon SM, Stamm WE, eds. *Sexually Transmitted Diseases*, Second edition, McGraw–Hill, Inc, 1990:547–60
20. The Vaginitis Report, National Vaginitis Association, Editor: Richard Sweet, MD, Fall 1996
21. Food and Drug Administration's (FDA) Clinical Chemistry and Clinical Toxicology Devices Panel Meeting December 7, 1999 (convened to discuss home use of vaginal pH testing) Transcript. Dr. Jane Schwebke, MD, FACP – Associate Professor of Medicine and Epidemiology, Medicine/Infectious Diseases and School of Public Health, University of Alabama at Birmingham, who was asked by FDA to speak as an expert, stated "I confess that I do pH's on all women I see . . ."
22. SAS Institute, Inc. Cary, NC, USA, 2000
23. Cytel Software Corp, Cambridge, MA, USA, 2000
24. Galen RS, Gambino SR. *Beyond Normality: The Predictive Value and Efficiency of Medical Diagnoses.* John Wiley and Sons, Inc, 1975:124
25. Weisberg M, Summers P. Patient self-diagnosis of vulvovaginal candidiasis. *Female Patient* 1996;21: 60–4
26. Ross RA, Lee M-LT, Onderdonk AB. Effect of Candida albicans infection and clotrimazole treatment on vaginal microflora *in vitro. Obstet Gynecol* 1995;86:925–30
27. Platz-Christensen JJ, Sundstrom E, Larsson P-G. Bacterial vaginosis and cervical intraepithelial neoplasia. *Acta Obstet Gynecol Scand* 1994;73: 586–8
28. Cohen CR, Duerr A, Pruithithada N, Rugpao S, Hillier SL, *et al.* Bacterial vaginosis and HIV seroprevalence among female commercial sex workers in Chiang Mai, Thailand. *AIDS* 1995; 9:1093–7
29. Hillier SL, Nugent RP, Eschenbach DA, Krohn MA, Gibbs RS, *et al.* Association between bacterial vaginosis and preterm delivery of low-birth-weight infants. *N Engl J Med* 1995;333:1737–42
30. Schwebke JR, Schulien MB, Zajackowski M. Pilot study to evaluate the appropriate management of patients with coexistent bacterial vaginosis and cervicitis. *Infect Dis Obstet Gynecol* 1995; 3:119–22
31. Sweet RL. Role of bacterial vaginosis in pelvic inflammatory disease. *Clin Infect Dis* 195;20 (Suppl 2):S271–5

Received 05/05/03; Accepted 09/25/03
